# The estimated burden of rare diseases in South Africa using Orphanet: an epidemiological analysis

**DOI:** 10.1186/s13023-026-04444-w

**Published:** 2026-06-11

**Authors:** Helen Louise Malherbe, Sujani Odendaal, Ana Kukava, Caterina Lucano

**Affiliations:** 1 Centre for Human Metabolomics, Desmond Tutu School of Medicine, Faculty of Health Sciences, North-West University, 11 Hoffmann Street, Potchefstroom, 2531 South Africa; 2 Independent Data Analyst, Roodepoort, Johannesburg, South Africa; 3 US14-Orphanet, INSERM (French National Institute for Health and Medical Research), 96 rue Didot, Paris, 75014 France

**Keywords:** Rare diseases, South Africa, Orphanet, Point prevalence, Burden of disease, Genetic diseases

## Abstract

**Background:**

Rare diseases (RDs) collectively affect a significant proportion of the population, yet their burden in South Africa (SA) remains poorly defined due to limited diagnostic capacity and infrastructure, inadequate epidemiological data and poor surveillance systems.

**Objective:**

To estimate the point prevalence of RDs in SA by integrating Orphanet-derived point prevalence data with national population statistics, identify disease characteristics and affected sub-groups, and assess the proportion of patients requiring high-cost treatments.

**Methods:**

A cross-sectional epidemiologic study was conducted using Orphanet’s validated methodology and four curated datasets (epidemiology, natural history, functional consequences, and medical domains). Inclusion criteria excluded cancers, infections, poisonings, and conditions without point prevalence data. RDs were classified by direct (population-based) or indirect (case/family report) point prevalence rates integrated with 2024 South African population estimates (*n* = 63015904). Additional analyses were performed for age of onset, inheritance patterns, medical domains, associated disability, and access to high-cost treatment access in SA.

**Results:**

A total of 3728 RD were included in the study, relating to 58% of unique Orphanet disorder entities. The estimated mean point prevalence of these RDs in SA was 4.8% (range: 3.7%–5.9%), corresponding to 3030204 (range: 2321906–3738503) affected individuals. Only 11% of RDs accounted for 98% of this patient burden. Most conditions had childhood onset (77%) and were inherited in an autosomal recessive (42%) or autosomal dominant (27%) pattern. Approximately 1.33 million (44%) patients in SA are estimated to experience moderate to severe functional disabilities related to the 3728 RD in the study. High-cost treatment was applicable to < 5% of patients for included RD, yet only 25 of 59 globally approved orphan drugs are currently available in SA.

**Conclusion:**

This study provides the first national-level, evidence-based estimate of RD prevalence in SA using global Orphanet data. The findings confirm a substantial, mostly paediatric burden, with significant disability and limited treatment access. Results support investment in newborn screening, integrated community genetic services, early diagnosis, and equitable care models. These estimates offer a critical foundation for evidence-based national RD policy development and service planning, and a replicable approach for other low- and middle-income countries.

**Supplementary Information:**

The online version contains supplementary material available at 10.1186/s13023-026-04444-w.

## Introduction

Rare diseases (RDs), while individually uncommon, collectively affect over 350 million people worldwide [1–3]. Defined by Rare Disease International (RDI) and the European Union (EU) as conditions affecting fewer than 1 in 2000 individuals, more than 80% of RDs have genetic or partially genetic causes, while others result from environmental exposures or infections [4–6]. Although most RDs are present from birth, clinical presentation may occur later. The RD collective is highly heterogeneous, currently comprising an estimated 6000 to 7000 distinct conditions [7]. A significant proportion of RD cases emerge in infancy or childhood and progress rapidly, with one-third of paediatric patients not reaching their fifth birthday [8].

People living with RD face a distinct set of diagnostic and health system challenges [9]. Globally, the average time to an accurate diagnosis exceeds five years, with longer delays in resource-constrained settings [10]. This diagnostic odyssey typically involves multiple misdiagnoses, unnecessary interventions, and substantial health system use with many patients remaining undiagnosed [11, 12]. Contributing factors include limited RD expertise among healthcare professionals (HCPs), insufficient diagnostic infrastructure, and poor access to coordinated care. Consequently, many patients experience premature mortality or preventable, unmitigated lifelong disability, poor quality of life, as well as social exclusion, marginalisation and stigma [9].

Therapeutic options remain limited, with only 5% of RDs having FDA-approved treatments [13]. However, some RD, such as phenylketonuria and congenital hypothyroidism, are manageable through early, low-cost interventions if diagnosed timeously - highlighting opportunities for timely diagnosis to improve outcomes and reduce long-term system burdens.

A fundamental barrier to addressing RD is the lack of epidemiological data [9]. The rarity of individual conditions also complicates clinical trial recruitment, real-world evidence generation, and surveillance. This results in a weak evidence base to guide policy and service planning. In evidence-based health systems, including that of South Africa (SA), accurate estimates of the RD burden are essential to inform priority-setting, resource allocation, and the design of equitable, responsive care models [9]. Estimating the prevalence of RD in SA is essential to support evidence-based policy and health system planning in a context where local data remain limited and fragmented.

Despite increasing international recognition of RD as a global health priority, as reflected in recent World Health Assembly (WHA) resolutions and other initiatives—most low- and middle-income countries (LMICs), including SA, lack national data to support targeted policy or programme responses [14–21]. Historical use of global prevalence estimates (6–8%) suggests that 3.6–4.8 million South Africans may be affected by RD [22, 23]. However, such estimates are insufficient for national planning, and locally derived data are required to move beyond advocacy to action [24].

Orphanet [25] is a comprehensive, curated international knowledge base for RD, integrating epidemiological, clinical, and functional data on RD nomenclature and classification, including > 6500 conditions. This study applies a validated methodology developed by Orphanet to generate national RD prevalence estimates for SA, stratified by demographic and clinical features, to support evidence-informed policy and health system response [1].

## Method

### Study design and overview

This study applied an epidemiologic method developed by Orphanet applied globally to estimate the national burden of RD in South Africa [1, 3].

The methodology integrated multiple, publicly available Orphanet datasets with national South African demographic data, applying consistent inclusion/exclusion criteria to define the eligible RD population. Further descriptive analyses characterized the resulting RD subset by epidemiologic, clinical, and functional features. High-cost therapy availability was explored through a supplementary expert-informed review.

### Data sources

#### Orphanet datasets

Four core datasets and supporting documentation were downloaded from Orphanet’s scientific knowledge portal [26] on 21 October 2024 (dataset release date: 31 July 2024) [7, 27–33]:

- Epidemiology of rare diseases.
- Natural history of rare diseases.
- Rare diseases and functional consequences.
- Linearisation of rare diseases.

Each Orphanet entry is uniquely identified by an ORPHAcode, a stable numeric identifier enabling linkage across datasets. These data were imported into Microsoft Power BI (v2.137.1102.0, October 2024) for cleaning and integration and then exported to Microsoft Excel for further processing and analysis alongside South African population data.

#### South African population data

National population estimates for 2024 (*n* = 63015904) were obtained online from Statistics South Africa [23].

### Inclusion and exclusion criteria

To ensure methodological consistency and prevent duplication, filters were applied to the integrated Orphanet dataset as previously used [1, 3]. Specific criteria used:

- **Entity type**: Only individual RD entities classified as “disorders” were included. Groups of disorders and subtypes were excluded.
- **Medical domain**: Disorders primarily categorized as rare neoplastic diseases, toxicologic disorders or infectious diseases were excluded.
- **Prevalence data**: Only disorders with reported point prevalence values were retained. Those described by annual incidence, birth prevalence or lifetime prevalence were excluded.
- **Prevalence threshold**: RDs were included only if their point prevalence was ≤ 1 in 2000. Those with a prevalence of 5–9 per 10000, > 1 per 1000, unknown or not yet documented were excluded.
- **Geographic scope**: Where multiple geographic estimates existed, a single prevalence value or range was selected using a ranked hierarchy: (1) Worldwide; (2) Europe; (3) European country; (4) United States; (5) Other continent; (6) Other country.

### Prevalence estimation

#### Direct point prevalence (value or class-based prevalence)

Point prevalence is recorded in Orphanet in one of two formats: a numeric value (“ValMoy”) or prevalence class. Where only class-based data were available (e.g. 1–9 per 1000000), standard mean, maximum and minimum values were assigned based on Orphanet conventions (Supplementary File 1: Point Prevalence Data Types). To avoid duplication, each disorder was assigned only one point prevalence value to be used in this study—either directly reported or derived.

Total *direct* point prevalence was calculated by summing these values across included RD and converted to patient (case) estimates using South Africa’s 2024 population:

$$\begin{gathered} \:Number\:of\:South\:African\:patients = \hfill \\ Direct\:Point\:Prevalence\: \times \:\frac{{South\:African\:Population}}{{100000}} \hfill \\ \end{gathered} $$

#### Indirect point prevalence (case and family reports)

For disorders without formal prevalence studies, Orphanet reports case/family counts from the literature. These were grouped and used to calculate an *indirect* point prevalence, following a method established in prior work [1]. The total number of global cases and families (multiplied by 10 to allow for varying family size) was divided by the 2024 global population of 8.2 billion [34].

$$\begin{gathered} \:Global\:indirect\:point\:prevalence = 100000\hfill \\ \: \times \:\frac{{\sum \: of\:cases\:and\:families\:\left( { \times 10} \right)\:reported}}{{Global\:Population}} \hfill \\ \end{gathered} $$

This global indirect point prevalence was then applied to the South African population to estimate the number of patients in South Africa using the previously outlined method (above) for the direct point prevalence.

#### Total point prevalence estimate

Direct and indirect prevalence values were combined to calculate the total estimated RD burden in South Africa, stratified by point prevalence range and presented per 100000 of the population. For formulae and calculations see Supplementary File 1: Point Prevalence Data Types.

### Descriptive analyses

The estimated patient population was further analysed using linked Orphanet datasets to describe key attributes:

- **Age of onset**: Disorders categorized by age at first clinical manifestation, based on Orphanet’s predefined age bands (antenatal to elderly) [30]. Age of onset was analysed descriptively and does not reflect disease duration or survival.
- **Type of inheritance**: Inheritance patterns were identified and summarized [30].
- **Medical domains**: Each RD was linked to one of Orphanet’s 25 included primary medical domains [32].
- **Functional consequences**: Impact on functional ability, including severity [31].

### Identification of high-cost drug-treatable RDs

A supplementary exercise was undertaken to identify RDs within the study subset with approved high-cost therapies:

- A desktop review was conducted in early 2025 to identify key peer-reviewed publications [13, 35]; global and local regulatory websites were used to identify high-cost drug–treatable conditions [36, 37] .
- RDs in the study subset were included if therapies were approved by at least one major regulatory body globally.
- A preliminary list of these RD was circulated among key RD stakeholders, including the National Bioproducts Institute [38], the South African Rare Disease Access Initiative [24], and the Interdisciplinary Scientific Committee of the International Rare Disease Research Consortium [39], for feedback and validation.
- The final list was compiled into a resource (Excel Worksheet) for continuous updating and national planning.

High-cost therapies were defined as products developed for small patient populations, typically with high development and unit costs.

## Results

### Study population and data characteristics

**Fig. 1:**
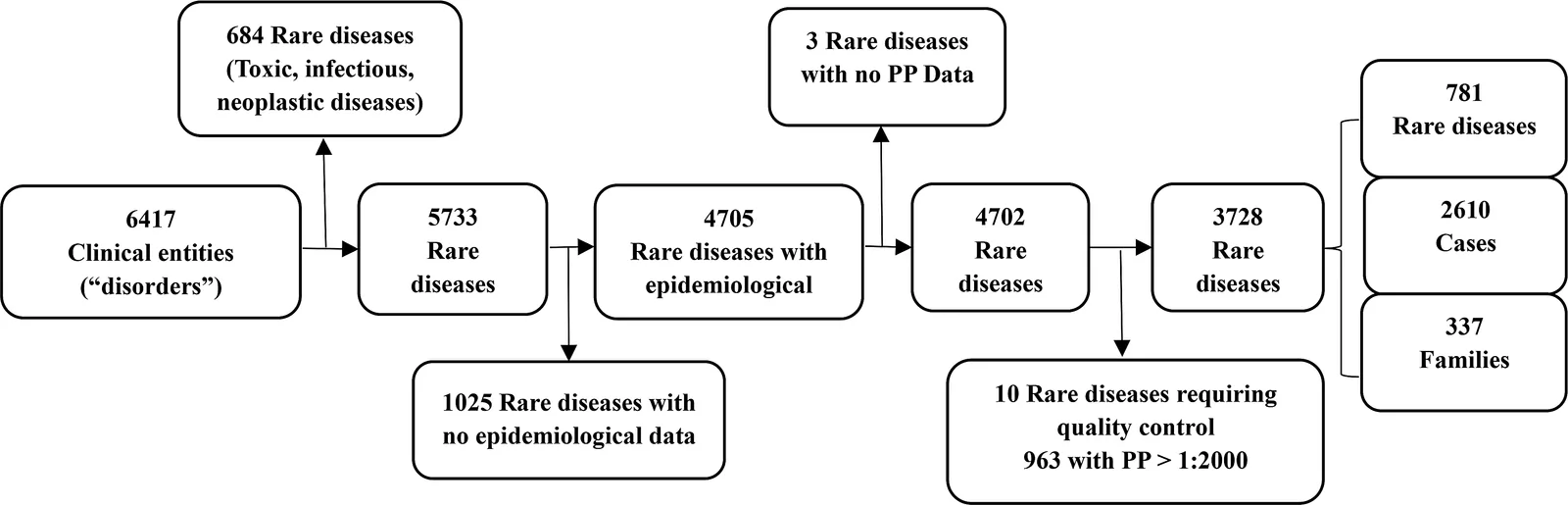
Selection process of point prevalence data from Orphanet’s epidemiological file used for analysis

A total of 6147 clinically unique RD (“disorder”) entities were identified in Orphanet. After all filters (inclusion/exclusion criteria) were applied (Fig. 1), 3728 RD (58%) remained, including 781 described with value or class-based prevalence values and 2947 described by cases (*n* = 2610) and family reports (*n* = 337).

### Point prevalence estimates

The total point prevalence of RD was 4.8% (range: 3.7% to 5.9%) translating to a mean of 3030204 (range: 2321906 to 3738503) affected by RDs in SA for the 3728 RD included in the study, see Tables 1 and 2.

**Fig. 2:**
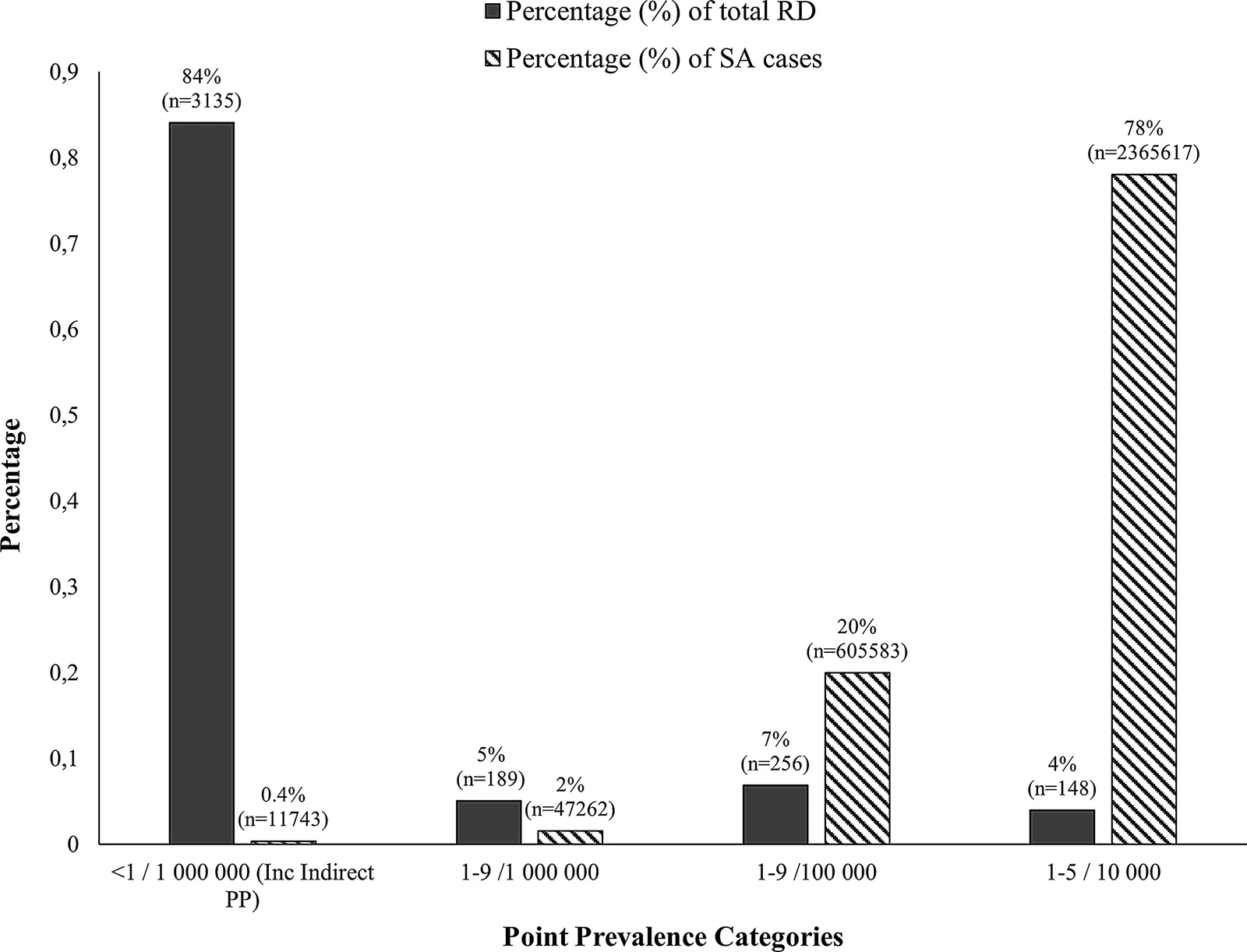
Distribution of rare diseases (proportion and number) and South African patients (proportion and number) for 3728 included conditions across point prevalence categories

Figure 2 charts the prevalence data across prevalence categories. A small proportion (11%, *n* = 404) of the most common RD account for 98% (*n* = 2971200) of the South African patient population. Conversely, 3135 (84%) of RD with a prevalence of ≤ 1 per 1000000, account for < 1% (*n* = 11743) of RD patients in the country. See Supplementary File 2: Key Data of Included Rare Diseases.

**Table 1:** Structure of rare disease dataset extracted from Orphanet

Category	Rare diseases (*n*)	Proportion (%)
**Indirect point prevalence**		
Cases	2610	70%
Families	337	9%
**Subtotal: Indirect**	**2947**	**79%**
**Direct point prevalence**		
Value & class (ValMoy)	433	12%
Class only	348	9%
**Subtotal: Direct**	**781**	**21%**
**Total**	**3728**	**100%**

**Table 2:** Estimated prevalence of rare diseases (global and South Africa)

Category	Estimated patients (Global)	Estimated patients (South Africa)	Prevalence (per 100,000 population)
**Indirect point prevalence** ^**a)**^			
Cases	99795	767	1.22
Families	34230	263	0.42
**Subtotal: Indirect**	134025	1030	1.63
**Direct point prevalence** ^**b)**^			
Value & class (ValMoy)	261252000	2007687	3186
Class only (minimum)	40754000	313189	497
Class only (mean)	132922000	1021488	1621
Class only (maximum)	225090000	1729787	2745
**Total Prevalence (minimum)**	302140025	2321906	3685
**Total Prevalence (mean)**	394308025	3030204	4809
**Total Prevalence (maximum)**	486476025	3738503	5933

Notes: (a) Indirect point prevalence (number of families × 10 to account for changing family size); (b) Total number of patients divided by the global population (8200000000) and resulting rate per 100000 used to calculate number of patients for South African population (*n* = 63015904) (23)

### Age of onset distribution

**Fig. 3:**
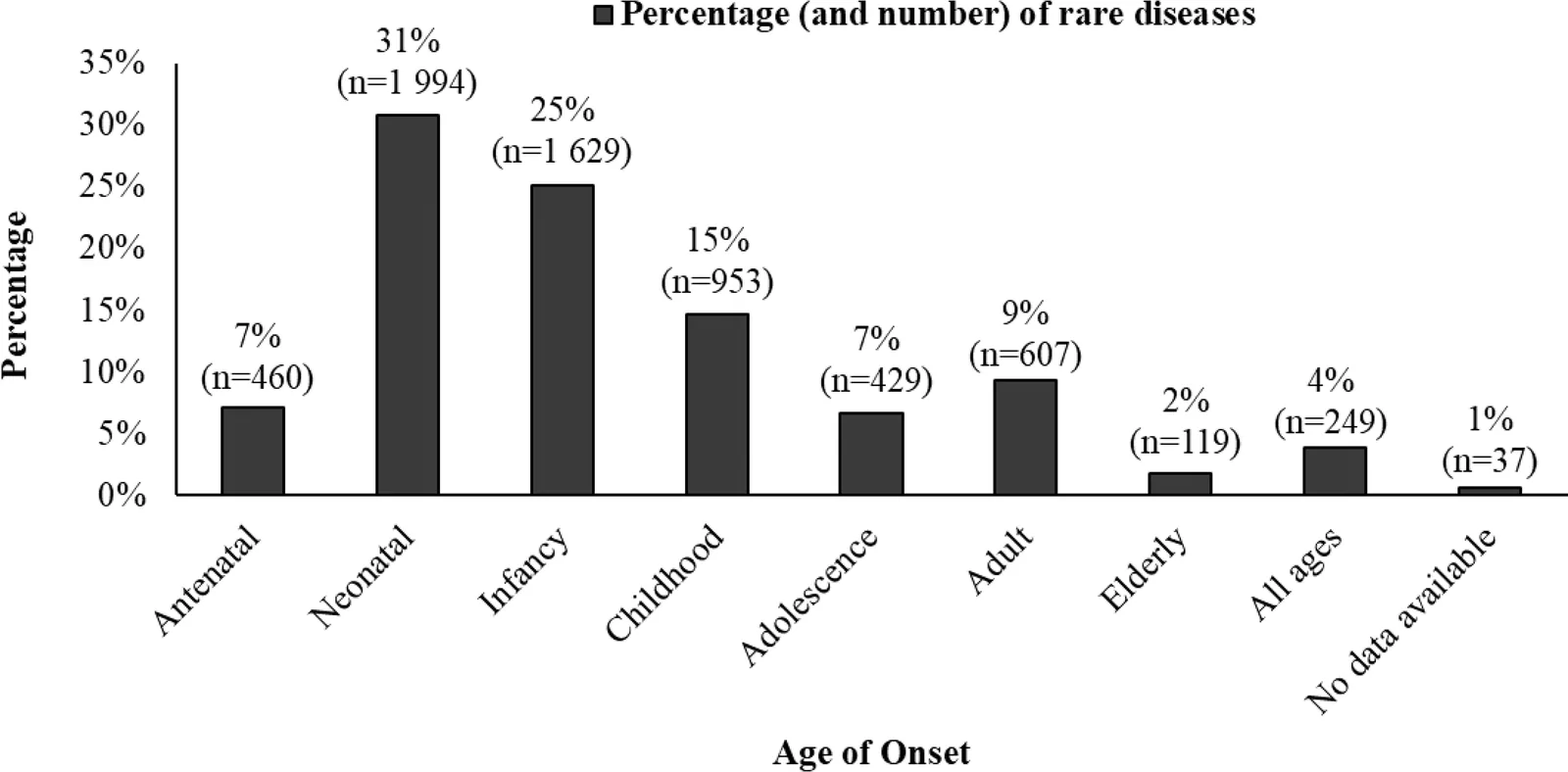
Age of onset for 3728 included rare diseases across the life course; age categories are not mutually exclusive. Antenatal: Before birth; Neonatal: 0–4 weeks; Infancy: 5 weeks − 23 months; Childhood: 2–11 years; Adolescent: 12–18 years; Adult:19–65 years; Elderly: >65 years; All ages: across life course with no peak; No data available: No information on the age of onset available

RDs included in the study were classified by their reported age of onset using Orphanet ontology (40), with the majority (31%) of conditions manifesting during the neonatal period (0–4 weeks of life). Specific patterns of onset varied significantly (Fig. 3), with 77% of disorders occurring during childhood (0–18 years) and 11% in adulthood (≥ 19 years).

### Inheritance patterns

**Fig. 4:**
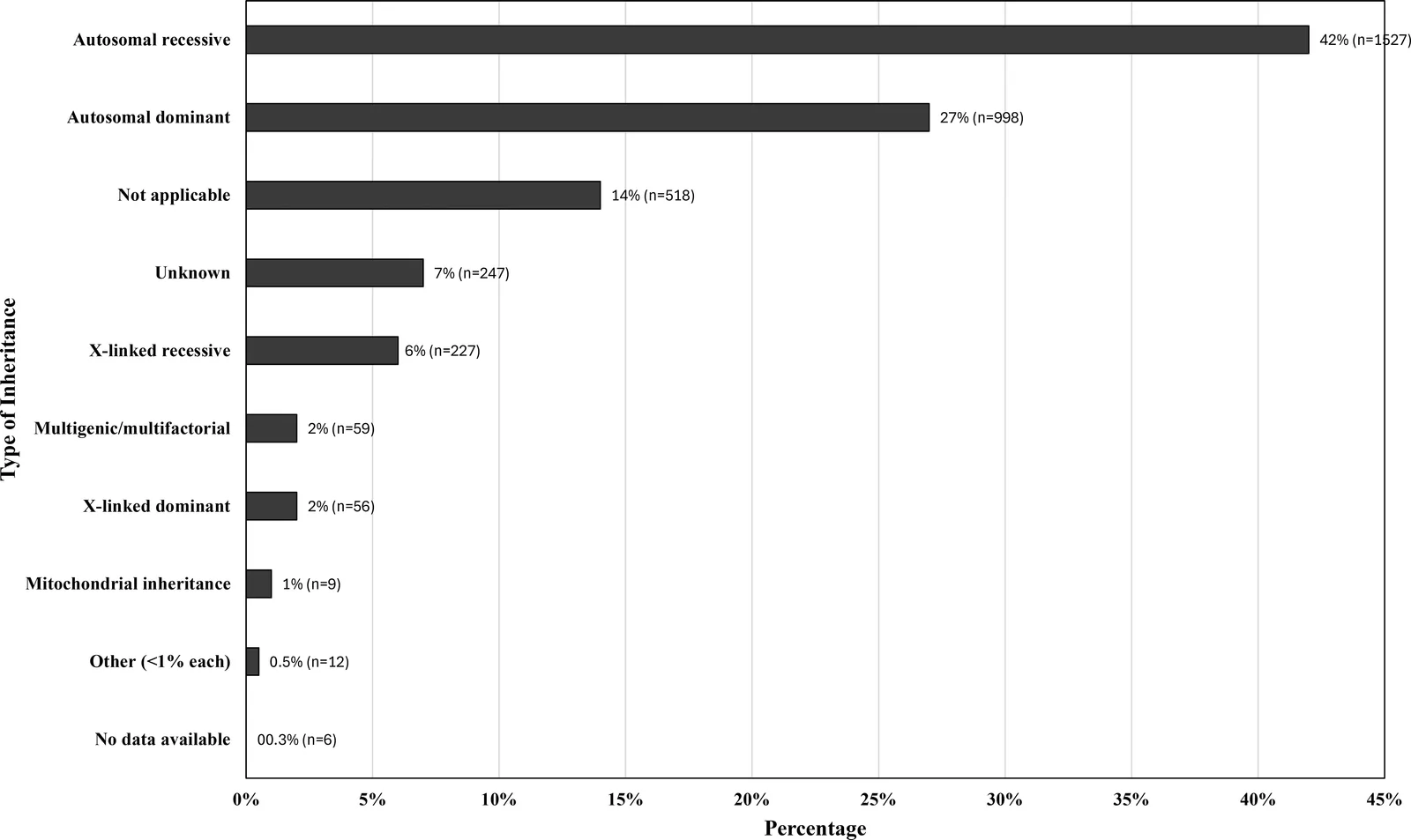
Distribution (percentage) of inheritance patterns of 3728 included rare diseases. Note: Categories are not mutually exclusive; Other (< 1%) = Oligogenic, Y-linked and Semi-dominant inheritance

Of the 3728 RD included, 99% had at least one inheritance pattern specified (Fig. 4). Most common were autosomal recessive (42%) and autosomal dominant (27%), and least common were semi-dominant oligogenic, and Y-linked inheritance. Refer to Orphanet ontology documentation for details on inheritance categories (40).

### Medical domain classification

Of the 25/28 Orphanet medical domains included, the two most common categories were rare developmental defects occurring during embryogenesis (37%) and rare neurological diseases (20%), based on both the number of RD, and the number and proportion of associated patients (cases), see Table 3. For the cadres of South African clinical specialties and allied HCP required per medical domain, see Supplementary File 3: Health Care Professional Cadres Required per Medical Domain.

**Table 3:** Distribution of rare disease patients and diseases across primary medical domains in South Africa, ranked by patient numbers (*n* = 3728 conditions)

Primary medical domain	Patients (*n*)	Patients (%)	Rare diseases (*n*)	Rare diseases (%)
**Rare developmental defects ** **during embryogenesis**	**733746**	**24%**	**1389**	**37%**
**Rare neurologic disease**	**361100**	**12%**	**752**	**20%**
**Rare systemic or ** **rheumatologic disease**	**265** **743**	**9%**	**109**	**3%**
Rare hematologic disease	195981	6%	116	3%
Rare respiratory disease	184433	6%	34	1%
Rare gastroenterologic disease	184080	6%	48	1%
Rare ophthalmic disorder	177133	6%	126	3%
Rare endocrine disease	173145	6%	109	3%
Rare skin disease	151470	5%	219	6%
Rare inborn errors of metabolism	128343	4%	286	8%
Rare cardiac disease	86847	3%	24	1%
Rare gynecologic/obstetric disease	76882	3%	5	< 1%
Rare renal disease	68887	2%	44	1%
Rare bone disease	60405	2%	271	7%
Rare hepatic disease	57013	2%	23	1%
Rare otorhinolaryngologic disease	30695	1%	21	1%
Rare immune disease	29593	1%	120	3%
Rare urogenital disease	22056	1%	3	< 1%
**Other (< 1% each)** ^**a**^	42955	1%	29	< 1%
**Total**	3030509	100%	3728	100%

^a^: Categories with < 1% patients include: rare odontologic disease; rare infertility, rare circulatory system disease, rare disorder potentially indicated for transplant/post-transplant complication, rare genetic disease, rare maxillo-facial surgical disease, rare abdominal surgical disease

### Functional consequences and disability

**Fig. 5:**
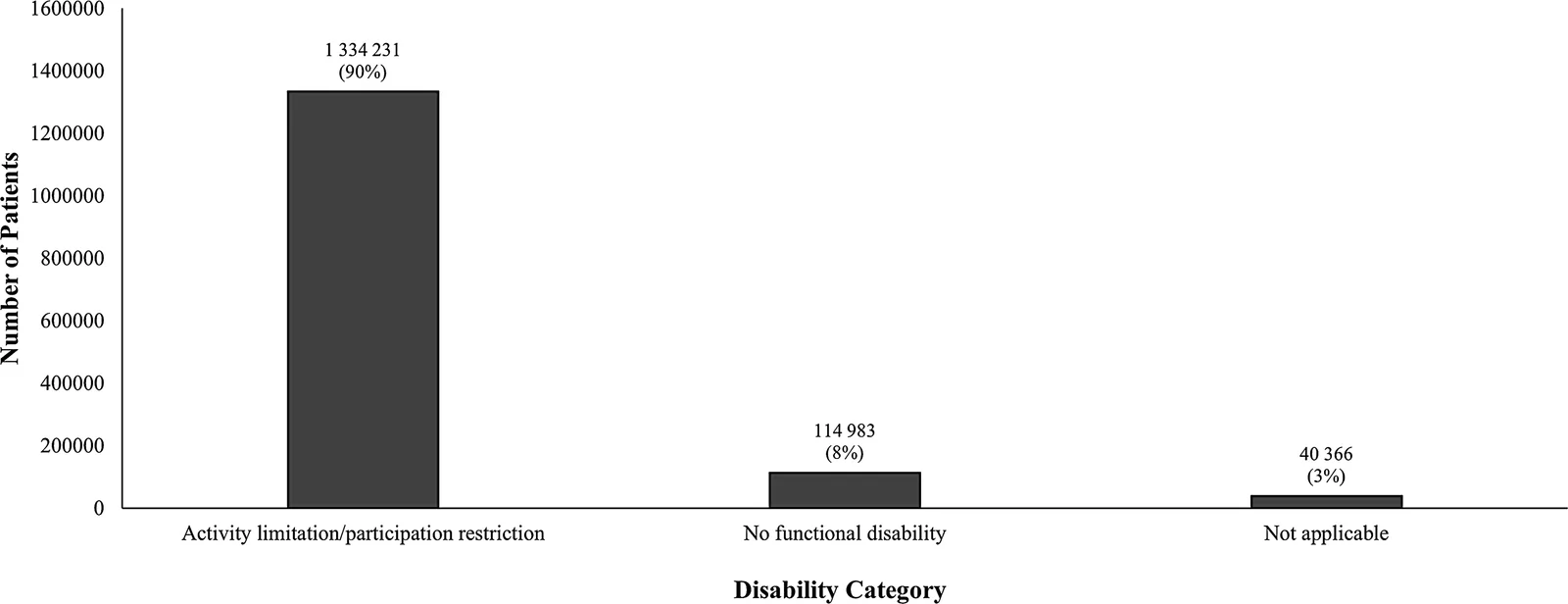
Estimated mean number and proportion (%) of patients in South Africa based on 569 (15%) rare diseases in Orphanet with associated disability data. Note: Not applicable includes: (1) Premature death within a year of onset/diagnosis; (2) Hypervariable functioning (abilities vary over time/situationally); (3) Other reasons

Information on functional consequences was available for 15% (*n* = 569**)** of the 3728 RD included (Fig. 5). Of these RD disability-affected patients, 90% (*n* = 1334231) had associated moderate to severe disability, and 8% (*n* = 114983) with no loss of ability.

### High-cost drug usage

A preliminary list comprising 59 RD was identified from the subset of 3728 Orphanet entries included in the study - with at least one high-cost drug approved by regulatory authorities globally. This equates to 150979 RD patients in SA requiring high-cost drugs.

Of the 57 globally approved therapeutics for these 59 RD, only 25 are registered in SA by the South African Health Products Regulatory Authority (SAHPRA) [36]. Of these 25 registered products, only a portion are available/accessible to patients. Unregistered products may be accessed through Section 21 of the Medicines and Related Substances Act, 1965 (Act No. 101 of 1965) [40], following an application to SAHPRA by individual HCP for a specific patient/group of patients. Examples of products being accessed via Section 21 are Zolgensma (Onasemnogene abeparvovec) for proximal spinal muscular atrophy (SMA) and Trikafta (Elexacaftor/tezacaftor/ivacaftor) for cystic fibrosis (CF) patients with eligible genotypes.

Categorised by Orphanet medical domains, most of the included RD requiring high-cost drugs were inborn errors of metabolism (*n* = 14), followed by rare immune diseases (*n* = 10), rare neurologic diseases (*n* = 9) and rare hematologic diseases (*n* = 8). See Supplementary File 4: List of High-Cost Drugs Approved/Available in SA for details.

## Discussion

This study aimed to provide more granular estimates of the burden of RD in SA, where observed epidemiological data are limited, and health priorities focus on more easily diagnosed and treated conditions. In the absence of national data, SA—like many LMICs—has relied on global estimates indicating that RD affect 6–8% of the population [1]. While useful as a general benchmark, these figures provide limited direction for policy or service planning. Our findings, using adapted Orphanet methodology, indicate that 4.8% (range: 3.7–5.9%) or 30302024 (range: 2321906–3738503) of South Africans may be affected by a subset of 3728 RD, closely aligning with recent global estimates [1]. Differences from earlier global Orphanet estimates [1] likely reflect the evolving and expanding nature of the Orphanet database over time, rather than true regional variation alone.

While broader global estimates of disease burden are available from sources such as the Global Burden of Disease study [41] and WHO Global Health Estimates [18, 42], these are based on different modelling approaches, data sources and disease classifications, and do not capture the full spectrum of RD. As such, they are not directly comparable to Orphanet-based cumulative point prevalence estimates.

A key study insight is the highly skewed distribution of disease burden, with a small subset of conditions accounting for the majority of affected individuals. While this pattern is consistent with global observations, its expression in SA is likely shaped by context-specific factors, including the country’s young population structure, high levels of genetic diversity, and variable access to diagnostic services. These factors may influence both the detection and survival of individuals with RD, and therefore the apparent distribution of burden. In addition, the analysis includes only 58% of unique RD available in Orphanet, which may further contribute to an underestimation of the full spectrum of disease burden. From a health system perspective, this concentration of burden within a limited number of conditions provides a pragmatic opportunity to prioritise service development, while recognising that the broader spectrum of RD remains under-identified and underserved.

Consistent with prior research, the majority (77%) of RD manifest in childhood [1]. These findings reflect patterns of clinical presentation in childhood and should not be interpreted as indicating disease duration or survival outcomes but rather emphasize the importance of early diagnosis and referral systems. This is particularly critical in SA, where 40% of the population is < 20 years of age, and over a million births occur annually [23, 43]. Strengthening antenatal and paediatric care pathways—including trained personnel and diagnostic infrastructure—is essential to address the needs of these vulnerable populations.

Inheritance patterns observed—mainly autosomal recessive and dominant—reinforce the need for strengthened community genetics services. The existing total capacity of skilled HCP in SA, in community genetics and other specialised clinical cadres, is completely inadequate [44, 45]. Moreover, the multidisciplinary care many RD patients require is largely unavailable or fragmented in SA, leaving most patients undiagnosed and untreated, often resulting in preventable early mortality and poor quality of life. Current ICD-10 coding also inadequately reflects the complexity of RD, further complicating surveillance and health system response in cases when a diagnosis is reached [46].

Disability is a major outcome of RD: for the 15% (*n* = 569/3728) of RD with associated disability data, > 90% (*n* = 1334231) of affected individuals, many of them children, experience functional impairment. Early diagnosis and access to rehabilitative therapies (e.g., speech, feeding, occupational, and physical therapy) can mitigate this burden. Similarly, high mortality linked to many RD highlights the urgent need for palliative care services that extend beyond oncology and HIV to include paediatric RD patient populations.

While high-cost treatments dominate RD discourse, this study indicates that < 5% (*n* = 150979) of RD patients for included RD would require such therapies. While further analyses are required to estimate more accurate patient numbers for each therapeutic incorporating specific RD age related prevalence, age of onset, age-stratified population groups and survivorship - these population level estimates highlight that such high-cost treatments are only applicable for a small proportion of RD patients.

Many of these treatments remain inaccessible, despite being approved or remain unaffordable in SA. While this challenges the narrative that all RD care is prohibitively expensive, it underscores the need for broader access to essential, low-cost interventions.

### Limitations

The study used Orphanet data and methodology, which includes only conditions classified as rare in Europe. RD due to infections, toxins, or rare cancers—relevant in LMIC contexts—were also excluded; Only RD with an assigned point prevalence value or range were included. Collectively these, and other criteria applied limited the study to 58% of total Orphanet RD entries. Age of onset was analysed descriptively and does not reflect disease duration or survival. The analysis did not incorporate age-stratified population structure or country-specific mortality rates, which may further influence these estimates in the South African context. Moreover, population prevalence as a measure can underestimate the burden of early-onset RD with high mortality, particularly where diagnosis is delayed or unavailable. Birth prevalence may therefore provide a more appropriate metric for capturing the true burden of early onset conditions and should be explored in future work.

A further limitation is the extrapolation of data from high-income countries to the South African setting, where diagnostic access, care pathways, and mortality profiles differ significantly. These estimates therefore reflect a “potential” burden under ideal care conditions and must be supplemented by local empirical studies.

## Conclusion and recommendations

This study provides the first national-level, data-driven estimate of the RD burden in SA, laying the groundwork for better policy and planning. The findings underscore the urgent need to scale up community genetics and integrated RD care services. Addressing RD requires investment, but the current lack of care carries significant socioeconomic and personal costs.

Several policy and research directions emerge:

1. Use of birth prevalence and age-stratified modelling to better estimate early-onset RD, particularly those with high infant mortality, incorporating age-specific population structure, disease duration, and country-specific survival and mortality rates to generate more contextually accurate burden estimates.
2. Provincial-level burden assessments to support localized planning.
3. Health economic evaluations, including the societal cost of delayed and/or no care.
4. Equity mapping of access across public vs. private sectors, and rural vs. urban areas.
5. Policy audits to identify gaps in access to diagnostics, treatments, and workforce development.
6. Local validation of global estimates via RD registries and clinical surveillance.

Addressing RD comprehensively will advance child survival goals and contribute to achieving 2030 Sustainable Development Goal 3 targets and alignment with the global universal health care agenda [47]. The findings support the implementation of proven interventions such as newborn screening, integrated community genetics, and better palliative and rehabilitative services.

## Supplementary Information

Below is the link to the electronic supplementary material.

Supplementary Material 1

Supplementary Material 2

Supplementary Material 3

Supplementary Material 4

## Data Availability

The datasets described in this article can be freely and openly accessed at the Orphadata Science platform (https://sciences.orphadata.com/) [48] and archived in a dedicated GitHub repository (https://github.com/Orphanet/Orphadata_aggregated) [49].
