# Supplementary material for: The estimated burden of rare diseases in South Africa using Orphanet: an epidemiological analysis
Source: Orphanet J Rare Dis. 2026 Jun 11;21:287. doi: 10.1186/s13023-026-04444-w (PMC13487910; doi:10.1186/s13023-026-04444-w)
Supplement: Supplementary file 1 [file 13023_2026_4444_MOESM1_ESM.pdf]

Point Prevalence Data Type

The point prevalence descriptors in Orphanet include three data types:

1) **Class and Value**: For RD with both a numerical point prevalence value and a prevalence range, the "ValMoy"[1] value in the Orphanet dataset (reported per 100 000 population) was used as the minimum, maximum and mean value.

2) **Class only**: For RD with a prevalence range, a minimum, maximum and mean value were assigned, specifically:
   a. <1 per 1 000 000: Minimum, maximum and mean all assigned as 0.1 per 100 000.
   b. 1-9 per 1 000 000: Minimum = 0.1 per 100 000; maximum = 0.9 per 100 000; mean = 0.5 per 100 000.
   c. 1-9 per 100 000: Minimum = 1 per 100 000; maximum = 9 per 100 000; mean = 5 per 100 000.
   d. 1-5 per 10 000: Minimum = 10 per 100 000; maximum = 50 per 100 000; mean = 30 per 100 000.

To prevent duplication, only one value was included per disorder (ORPHAcode), either from class and value (ValMoy), or class only (mean) as described in 1) and 2) above.

Resulting values from these two types of data (class and value, and class only) were then summed to calculate the direct point prevalence per 100 000 population.

This direct point prevalence was then used to calculate the number of South African patients using the total population (63 015 904) (23), as follows:

$$Number\ of\ South\ African\ patients = Direct\ Point\ Prevalence\ x\ \frac{South\ African\ Population}{100\ 000}$$

3) **Case and family reports:** For RD where there is no population-based study, Orphanet collects the number of cases or families from published case report(s), case series or recent review of the worldwide literature - and calculates the total number of cases or families reported (37). The lowest point prevalence range (<1/1,000,000) is assigned to the disorder.

   In this study, for RD described by case or family reports, individual point prevalence values were not calculated. Instead, these cases were tackled as a group to calculate an indirect point prevalence, as previously described by Orphanet (1, 2, 34). To minimize the effect of changing family size this analysis was repeated using ten cases per family. Specific steps included:

   a. The global indirect point prevalence was first calculated by summing the total number of cases (ValMoy[6]) reported by cases/families, divided by the global population (8 200 000 000).

[1] ValMoy is the mean value of a given prevalence type in Orphanet.

$$Global\ indirect\ point\ prevalence = 100\,000\ x\ \frac{\sum\ \ of\ cases\ and\ families\ (x10)\ reported}{Global\ Population}$$

b. The resulting global indirect point prevalence rate per 100 000 population was then applied to the 2024 South African population (63 015 904) (23) to calculate the number of patients in South Africa using the previously outlined method (above) for the direct point prevalence.
